# Supplementary material for: Characterization of Anaerobic Rumen Fungal Community Composition in Yak, Tibetan Sheep and Small Tail Han Sheep Grazing on the Qinghai-Tibetan Plateau
Source: Animals (Basel). 2020 Jan 16;10(1):144. doi: 10.3390/ani10010144 (PMC7023293; doi:10.3390/ani10010144)
Supplement: Supplementary file 1 [file animals-10-00144-s001.pdf]

Supplementary Materials

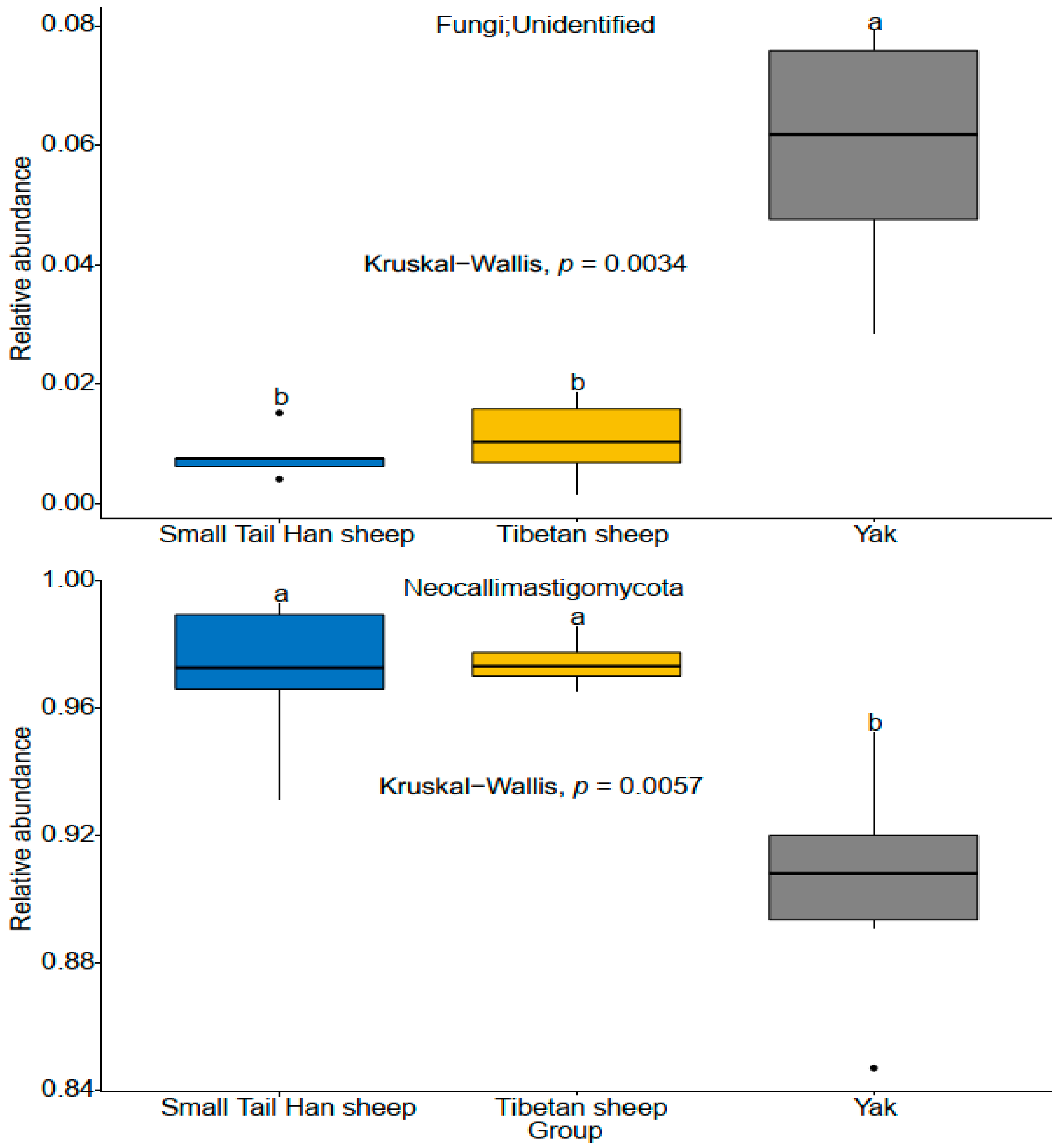

Figure S1. The significantly different phyla among three ruminant species. The Wilcoxon test was used to determine the significant level between two animal groups, while Kruskal-Wallis test was used to calculate the significant level among three animal groups. a, b Different letters indicate a significant difference between animal species ($P < 0.05$).

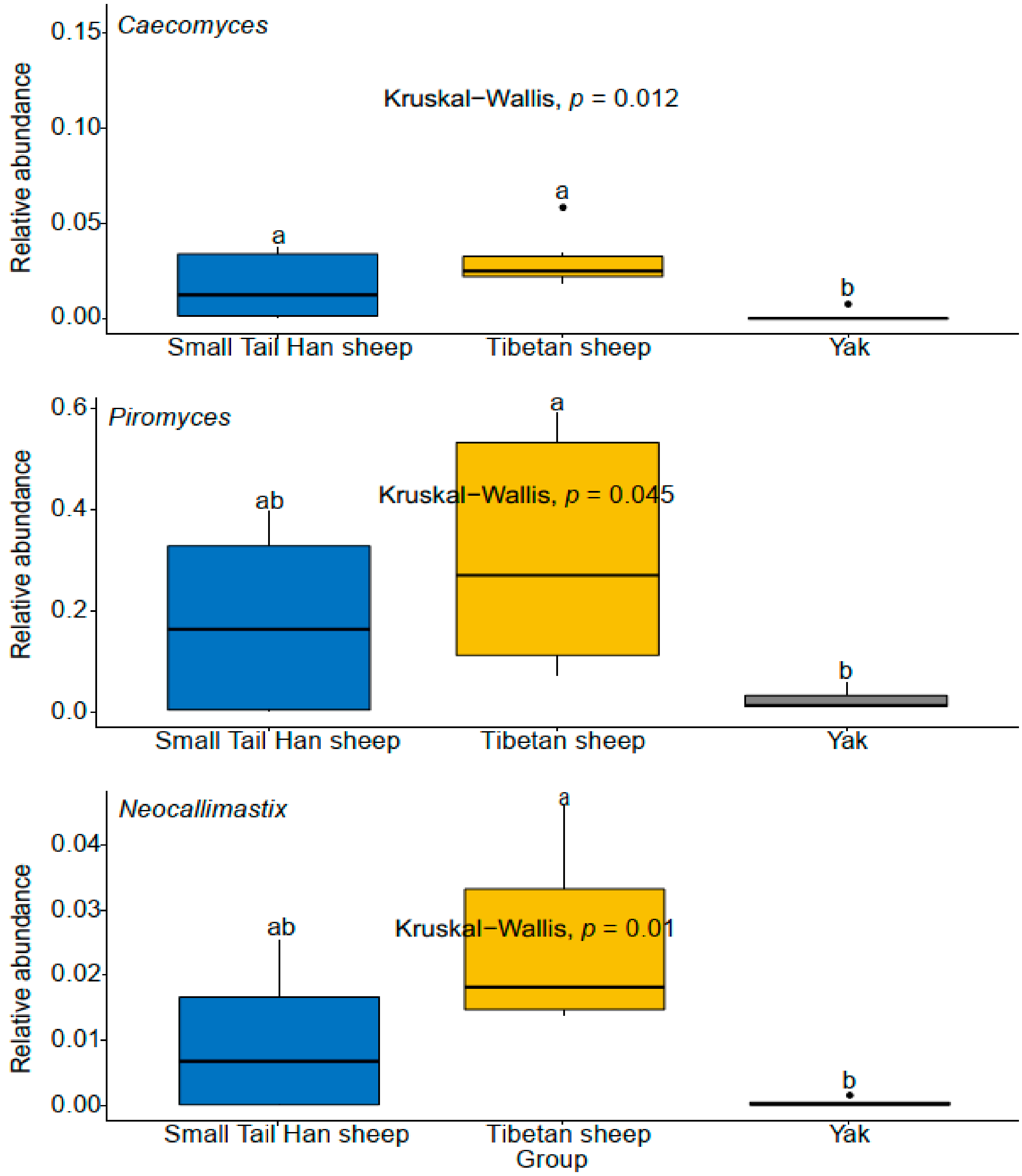

Figure S2. Genera with significant differences in three ruminant species. The Wilcoxon test was used to determine the significant level between two animal groups, while Kruskal-Wallis test was used to calculate the significant level among three animal groups. a, b Different letters indicate a significant difference between animal species ($P < 0.05$).

Table S1. The distribution of sequences and OTUs in all samples

Sample ID	Group	Sequence count	Number of OTU
Y7	Yak	84077	255
Y8	Yak	82390	226
Y9	Yak	84238	209
Y10	Yak	80127	180
Y11	Yak	84359	204
Y12	Yak	75078	187
Z104	Tibetan sheep	83878	157
Z106	Tibetan sheep	82135	138
Z109	Tibetan sheep	77128	200
Z113	Tibetan sheep	90305	131
Z121	Tibetan sheep	89161	177
Z123	Tibetan sheep	80350	173
X202	Small Tail Han sheep	95412	169
X208	Small Tail Han sheep	92178	185
X212	Small Tail Han sheep	77794	146
X216	Small Tail Han sheep	85010	232
X218	Small Tail Han sheep	89514	150
X221	Small Tail Han sheep	96619	108

Table S2. The topological features at each ruminant species

Taxa	node.degree	betweenness.centrality	closeness.centrality	node.transitivity
**Small Tail Han sheep**				
No Blast hit;Other	9	1.19	0.11	0.81
Anaeromyces	7	0.68	0.09	0.81
Caecomyces	8	0.88	0.10	0.82
Cyllamyces	8	0.88	0.10	0.82
Neocallimastix	7	0.68	0.09	0.81
Oontomyces	7	0.31	0.09	0.90
Orpinomyces	9	1.19	0.11	0.81
Piromyces	8	0.62	0.10	0.86
Neocallimastigaceae;unidentified	7	0.31	0.09	0.90
unidentified	6	0.29	0.08	0.87
**Tibetan sheep**				
No Blast hit;Other	7	0.33	0.11	0.90
Anaeromyces	8	0.48	0.13	0.89
Caecomyces	7	0.14	0.11	0.95
Cyllamyces	8	0.48	0.13	0.89
Neocallimastix	7	0.14	0.11	0.95

Orpinomyces	7	0.33	0.11	0.90
Piromyces	8	0.48	0.13	0.89
Neocallimastigaceae;unidentified	6	0.14	0.10	0.93
unidentified	8	0.48	0.13	0.89
**Yak**				
No Blast hit;Other	5	0.17	0.08	0.90
Anaeromyces	7	0.62	0.09	0.86
Caecomyces	7	0.37	0.09	0.90
Cyllamyces	6	0.00	0.08	1.00
Neocallimastix	8	0.98	0.10	0.82
Oontomyces	6	0.62	0.08	0.80
Orpinomyces	7	0.82	0.09	0.81
Piromyces	8	1.40	0.10	0.79
Neocallimastigaceae;unidentified	9	2.02	0.11	0.75
unidentified	9	2.02	0.11	0.75
